# Supplementary material for: Referral challenges and outcomes of neonates received at Muhimbili National Hospital, Dar es Salaam, Tanzania
Source: PLoS One. 2022 Jun 15;17(6):e0269479. doi: 10.1371/journal.pone.0269479 (PMC9200315; doi:10.1371/journal.pone.0269479)
Supplement: S1 File — (DOCX) [file pone.0269479.s001.docx]

## Questionnaire – English Version

**Demographic data:**

Serial No……………………… Date………………………. Date of birth ……/……/……….

Time of Birth ………… AM/PM

Date of referral …/……/ Date of admission…/……/…. Residence………………………

Time of admission…………………

**Neonate’s characteristics**

Gestational age in weeks …… Sex: Male Female

Birth weight …………………………kgs

Mode of delivery: SVD SBD AVD CS Not documented

Apgar score at 1min……. 5min……10 min…………. Not documented ……………………

**Referral information:**

Name of referring institution…………………………………………………………

Reason for referral

1. Lack of a newborn unit
2. Lack of equipment
3. For investigation
4. Lack of personnel
5. For specialized care
6. Others; specify………………………
7. Not documented ……….

Notification given to the Neonatal unit of MNH before transfer YES NO

Neonate escorted by any personnel YES NO

**Health professional escorting neonate**

1. Who escorted the neonate?

1. Nurse assistant
2. nurse attendant
3. Registered nurse
4. Family member
5. Doctor
6. Other, specify……………………………………

2. Have you been trained on essential newborn care? YES NO

3. If YES, Mention three components of essential newborn care

CIRCLE appropriately

1. Ensuring warmth
2. Immediate skin to skin care
3. Early breastfeeding
4. Umbilical cord care
5. Eye care
6. Vitamin K administration
7. immunization

4. Was any monitoring done during transport? YES NO

If yes, specify

1. Temperature monitoring Yes No
2. Monitoring of circulation Yes No
3. RBG monitoring Yes No
4. Monitoring of oxygen saturation Yes No

5. By which method was the baby kept warm during transport?

1. incubator
2. using blankets/warm clothes
3. kept close with the mother in a KMC position
4. polythene bags/sheets
5. Others………

6. Was the RBG checked before transfer? YES NO Don’t know

1. If YES, what was the value? ………..
2. If LOW what was done? …………..

7. Was the oxygen saturation checked before transfer?

1. YES
2. NO
3. don’t know
4. If YES what was the value in percentage? ……………
5. If LOW what was done? ……………….

8. Was the body temperature checked before transfer?

1. YES
2. NO
3. Don’t know
4. If YES what was the value in degree Celsius? …………….
5. If LOW what was done? ……………

9. Was an intravenous line secured before transfer?

1. YES
2. NO

**TRANSPORT CHARACTERISTICS**

10. What was the mode of transport?

1. Ambulance
2. Private car/taxi
3. Motor bike
4. Tricyclic motor vehicle
5. Public service vehicle

**CONDITION OF NEONATE AT ARRIVAL**

Temperature: ___________________degrees Celsius

Capillary refill time (CRT): ___________________seconds

Oxygen saturation (SPO2) __________________%

Random blood sugar (RBG): ___________________ mmol/l

## Data checklist

|  | **YES** | **NO** |
| --- | --- | --- |
| **Referral letter present**   If yes, is it complete? |  |  |
| **Did neonate require oxygen?**   If yes, was oxygen given? |  |  |
| **Intravenous line inserted** |  |  |
| **Was monitoring done en route of?**  **a) Temperature**  **b) Respiration**  **c) Circulation** |  |  |
| **Was the neonate kept warm?** |  |  |

**WHO Ambulance checklist**

|  | **YES** | **NO** |
| --- | --- | --- |
| **OXYGEN SUPPLY**   Was oxygen delivery intact |  |  |
| **Incubator present** |  |  |
| **Resuscitation equipment: (ambu bags)** |  |  |
| **Essential medicine i.e. adrenaline** |  |  |
| **Monitoring equipment e.g. thermometers, pulse oximeters** |  |  |
| **Intravenous fluids** |  |  |
| **Suctioning apparatus** |  |  |

**OUTCOME**

48 hours post admission

Survived

Died
