# Supplementary material for: Referral challenges and outcomes of neonates received at Muhimbili National Hospital, Dar es Salaam, Tanzania
Source: PLoS One. 2022 Jun 15;17(6):e0269479. doi: 10.1371/journal.pone.0269479 (PMC9200315; doi:10.1371/journal.pone.0269479)
Supplement: Attachment — Submitted filename: PONE-D-21-40535 (1).pdf [file pone.0269479.s003.pdf]

PLOS ONE

Referral Challenges and Outcomes of Neonates Received at Muhimbili National Hospital, Dar es Salaam, Tanzania.

--Manuscript Draft--

**Manuscript Number:**	PONE-D-21-40535
**Article Type:**	Research Article
**Full Title:**	Referral Challenges and Outcomes of Neonates Received at Muhimbili National Hospital, Dar es Salaam, Tanzania.
**Short Title:**	Referral Challenges and Outcomes of Neonates Received at Muhimbili National Hospital, Dar es Salaam, Tanzania.
**Corresponding Author:**	MPOKIGWA KIPUTA<br>Muhimbili University of Health and Allied Sciences<br>Dar es Salaam, TANZANIA, UNITED REPUBLIC OF
**Keywords:**	Neonate; Neonatal transportation; Neonatal mortality; Referral; admission clinical status.
**Abstract:**	Background<br><br>Functional referral system including pre referral care, access to emergency transport and ensuring continuity of care between facilities is critical for improved newborn health outcome. This study assessed the admission clinical status, transportation factors and outcomes of referred neonates at Muhimbili National Hospital (MNH), a tertiary facility in Dar es Salaam, Tanzania.<br><br>Methods<br><br>A descriptive cross sectional study with a longitudinal follow up was conducted from September 2020 to February 2021, including all neonates received at MNH. A structured questionnaire was used to collect demographic characteristics and transport factors were extracted from the referral documents and by interviewing caregivers or escorting person/nurse. Ambulances were directly observed using a structured checklist on presence, absence and functionality of supportive care during transportation. All enrolled neonates had a clinical assessment at admission and 48 hours post admission to determine 48 hours' clinical outcome as either survived/died.<br><br>Results<br><br>Out of the 348 neonates assessed during the study period, the median gestation age was 38 weeks (IQR 32, 39) with the mean birth weight of 2461 ± 936 g. Pre referral documentation showed that temperature was measured in 176 (57.1%), oxygen saturation and random blood glucose in only 143 (46.6%) and 116 (36.2%) neonates respectively. Ambulance was used as a means of transportation among 308 (88.5%) neonates. While no ambulance had an incubator only 7 (2.0%) neonates were kept on a Kangaroo Mother Care (KMC) position. Monitoring enroute was done to only 94 (27%) of the transferred neonates. On arrival, the clinical status of the neonates was as follows; 115 (33%) hypothermic, 74 (21.3%) hypoxic, 30 (8.6%) with poor perfusion and 49 (14.1%) hypoglycemic. Those with hypothermia (< 36.50C), were two times more likely to die compared to those normothermic neonates (OR=2.09, 95% CI (1.05-4.20), p=0.037). Thirty-eight (50.7%) of those who had hypoxia (SPO2 < 90%) at arrival died compared to 37 (49.3%) of those who were not hypoxic (OR=2.88, 95%CI (1.44-5.74), p=0.003). Twenty-one (67.7%) neonates with prolonged capillary refill time (CRT) died compared to ten (32.3%) who survived (OR=4.76, 95%CI (1.80-12.58), p=0.002). Out of forty-nine neonates who arrived with hypoglycemia (RBG < 2.5mmol/l) twenty-one (42.9%) died in the first 48 hours (OR=2.13, 95%CI (0.96-4.74), p=0.064). Additionally, neonates who had hyperglycemia (RBG > 8.3mmol/l) on arrival were three times more likely to die compared to those who were euglycemic [(OR= 3.10, 95% CI (1.19 – 8.09) p=0.021]. Overall mortality was 22.4% within 48 hours of admission and risk of dying increase as the presence of poor clinical status add on.

Powered by Editorial Manager® and ProduXion Manager® from Aries Systems Corporation

	Conclusion<br><br>Neonatal transportation in Dar es Salaam, Tanzania was observed to be challenging. Pre transfer care and monitoring during transportation was inadequate and this contributed to poor clinical status on admission. Hypothermia, hypoglycemia, hyperglycemia, hypoxia and poor perfusion on admission were associated with increased mortality.
**Order of Authors:**	MPOKIGWA KIPUTA<br>Nahya Salim<br>Peter P Kunambi<br>Augustine Massawe
**Additional Information:**	
**Question**	**Response**
**Financial Disclosure**<br><br>Enter a financial disclosure statement that describes the sources of funding for the work included in this submission. Review the submission guidelines for detailed requirements. View published research articles from *PLOS ONE* for specific examples.<br><br>This statement is required for submission and **will appear in the published article** if the submission is accepted. Please make sure it is accurate.<br><br>**Unfunded studies**<br>Enter: *The author(s) received no specific funding for this work.*<br><br>**Funded studies**<br>Enter a statement with the following details:<br>• Initials of the authors who received each award<br>• Grant numbers awarded to each author<br>• The full name of each funder<br>• URL of each funder website<br>• Did the sponsors or funders play any role in the study design, data collection and analysis, decision to publish, or preparation of the manuscript?<br>• **NO** - Include this sentence at the end of your statement: *The funders had no role in study design, data collection and analysis, decision to publish, or preparation of the manuscript.*<br>• **YES** - Specify the role(s) played.<br><br>* typeset	This study received financial support from the Government of Tanzania through the Ministry of Health, Community Development, Gender, Elderly and Children at Muhimbili University of Health and Allied Sciences, MUHAS. The funders had no role in study design, data collection and analysis, decision to publish, or preparation of the manuscript.

**Competing Interests**<br><br>Use the instructions below to enter a competing interest statement for this submission. On behalf of all authors, disclose any competing interests that could be perceived to bias this work—acknowledging all financial support and any other relevant financial or non-financial competing interests.<br><br>This statement is required for submission and **will appear in the published article** if the submission is accepted. Please make sure it is accurate and that any funding sources listed in your Funding Information later in the submission form are also declared in your Financial Disclosure statement.<br><br>View published research articles from *PLOS ONE* for specific examples.<br><br>**NO authors have competing interests**<br><br>Enter: *The authors have declared that no competing interests exist.*<br><br>**Authors with competing interests**<br><br>Enter competing interest details beginning with this statement:<br><br>*I have read the journal's policy and the authors of this manuscript have the following competing interests: [insert competing interests here]*<br><br>* typeset	The authors have declared that no competing interests exist
**Ethics Statement**<br><br>Enter an ethics statement for this submission. This statement is required if the study involved:<br><br>• Human participants<br>• Human specimens or tissue<br>• Vertebrate animals or cephalopods	Permission to carry out the study was sought from the Muhimbili University of Health and Allied Sciences (MUHAS) Institutional Review Board (IRB) with an approval number MUHAS-REC-07-2020-306. Written informed consent to participate in the study was sought from both the parent/guardian and health care personnel who escorted the neonate prior to any study procedure. Illiterate parents/ guardians were asked for a witness who participated within the discussion prior to obtaining their thumbprints and witness signature. All neonates received appropriate treatment according to the national treatment guidelines of Tanzania regardless of participation.

• Vertebrate embryos or tissues<br>• Field research<br><br>Write "N/A" if the submission does not require an ethics statement.<br><br>General guidance is provided below. Consult the submission guidelines for detailed instructions. **Make sure that all information entered here is included in the Methods section of the manuscript.**	

Powered by Editorial Manager® and ProduXion Manager® from Aries Systems Corporation

**Format for specific study types**<br><br>**Human Subject Research (involving human participants and/or tissue)**<br>• Give the name of the institutional review board or ethics committee that approved the study<br>• Include the approval number and/or a statement indicating approval of this research<br>• Indicate the form of consent obtained (written/oral) or the reason that consent was not obtained (e.g. the data were analyzed anonymously)<br><br>**Animal Research (involving vertebrate animals, embryos or tissues)**<br>• Provide the name of the Institutional Animal Care and Use Committee (IACUC) or other relevant ethics board that reviewed the study protocol, and indicate whether they approved this research or granted a formal waiver of ethical approval<br>• Include an approval number if one was obtained<br>• If the study involved *non-human primates*, add *additional details* about animal welfare and steps taken to ameliorate suffering<br>• If anesthesia, euthanasia, or any kind of animal sacrifice is part of the study, include briefly which substances and/or methods were applied<br><br>**Field Research**<br><br>Include the following details if this study involves the collection of plant, animal, or other materials from a natural setting:<br>• Field permit number<br>• Name of the institution or relevant body that granted permission	
**Data Availability**<br><br>Authors are required to make all data underlying the findings described fully available, without restriction, and from the time of publication. PLOS allows rare exceptions to address legal and ethical concerns. See the PLOS Data Policy and FAQ for detailed information.	Yes - all data are fully available without restriction

Powered by Editorial Manager® and ProduXion Manager® from Aries Systems Corporation

A Data Availability Statement describing where the data can be found is required at submission. Your answers to this question constitute the Data Availability Statement and **will be published in the article**, if accepted.<br><br>**Important:** Stating 'data available on request from the author' is not sufficient. If your data are only available upon request, select 'No' for the first question and explain your exceptional situation in the text box.<br><br>Do the authors confirm that all data underlying the findings described in their manuscript are fully available without restriction?	
**Describe where the data may be found in full sentences. If you are copying our sample text, replace any instances of XXX with the appropriate details.**<br><br>• If the data are **held or will be held in a public repository**, include URLs, accession numbers or DOIs. If this information will only be available after acceptance, indicate this by ticking the box below. For example: *All XXX files are available from the XXX database (accession number(s) XXX, XXX.).*<br>• If the data are all contained **within the manuscript and/or Supporting Information files**, enter the following: *All relevant data are within the manuscript and its Supporting Information files.*<br>• If neither of these applies but you are able to provide **details of access elsewhere**, with or without limitations, please do so. For example:<br><br>*Data cannot be shared publicly because of [XXX]. Data are available from the XXX Institutional Data Access / Ethics Committee (contact via XXX) for researchers who meet the criteria for access to confidential data.*<br><br>*The data underlying the results presented in the study are available from (include the name of the third party*	All relevant data are within the manuscript and its supporting information files

and contact information or URL).<br>• This text is appropriate if the data are owned by a third party and authors do not have permission to share the data.<br><br>* typeset	
Additional data availability information:	

Manuscript
Click here to access/download;Manuscript;Manuscript_Mpokigwa Kiputa_PLOS Medicine.docx

Referral Challenges and Outcomes of Neonates Received at Muhimbili National Hospital, Dar es Salaam, Tanzania

Referral challenges and Outcome of neonates

Mpokigwa Kiputa[1*¶], Nahya Salim[1*¶], Peter. P. Kunambi[2], Augustine Massawe[1‡]

[1]Department of Paediatrics and Child Health, Muhimbili University of Health and Allied sciences, Dar es Salaam, Tanzania.

[2]Department of Clinical Pharmacology, Muhimbili University of Health and Allied sciences, Dar es Salaam, Tanzania.

* Corresponding authors

E-mails: mpokigwakiputa@gmail.com (MK); nsalim@ihi.or.tz (NS)

¶ MK and NS contributed equally to this work

Funding: This study received financial support from the Government of Tanzania through the Ministry of Health, Community Development, Gender, Elderly and Children at Muhimbili University of Health and Allied Sciences, MUHAS. The funders had no role in study design, data collection and analysis, decision to publish, or preparation of the manuscript.

Competing interests: The authors have declared that no competing interests exist.

Abstract

Background: Functional referral system including pre referral care, access to emergency transport and ensuring continuity of care between facilities is critical for improved newborn health outcome. This study assessed the admission clinical status, transportation factors and outcomes of referred neonates at Muhimbili National Hospital (MNH), a tertiary facility in Dar es Salaam, Tanzania.

Methods: A descriptive cross sectional study with a longitudinal follow up was conducted from September 2020 to February 2021, including all neonates received at MNH. A structured questionnaire was used to collect demographic characteristics and transport factors were extracted from the referral documents and by interviewing caregivers or escorting person/nurse. Ambulances were directly observed using a structured checklist on presence, absence and functionality of supportive care during transportation. All enrolled neonates had a clinical assessment at admission and 48 hours post admission to determine 48 hours' clinical outcome as either survived/died.

Results: Out of the 348 neonates assessed during the study period, the median gestation age was 38 weeks (IQR 32, 39) with the mean birth weight of 2461 ± 936 g. Pre referral documentation showed that temperature was measured in 176 (57.1%), oxygen saturation and random blood glucose in only 143 (46.6%) and 116 (36.2%) neonates respectively. Ambulance was used as a means of transportation among 308 (88.5%) neonates. While no ambulance had an incubator only 7 (2.0%) neonates were kept on a Kangaroo Mother Care (KMC) position. Monitoring enroute was done to only 94 (27%) of the transferred neonates. On arrival, the clinical status of the neonates was as follows; 115 (33%) hypothermic, 74 (21.3%) hypoxic, 30 (8.6%) with poor perfusion and 49 (14.1%) hypoglycemic. Those with hypothermia (< 36.5^{0}C), were two times more likely to die compared to those normothermic neonates (OR=2.09, 95% CI (1.05-4.20), p=0.037). Thirty-eight (50.7%) of those who had hypoxia (SPO2 < 90%) at arrival died compared to 37 (49.3%) of those who were not hypoxic (OR=2.88, 95%CI (1.44-5.74), p=0.003). Twenty-one (67.7%) neonates with prolonged capillary refill time (CRT) died compared to ten (32.3%) who survived (OR=4.76, 95%CI (1.80-12.58), p=0.002). Out of forty-nine neonates who arrived with hypoglycemia (RBG < 2.5mmol/l) twenty-one (42.9%) died in the first 48 hours (OR=2.13, 95%CI (0.96-4.74), p=0.064). Additionally, neonates who had hyperglycemia (RBG > 8.3mmol/l) on arrival were

three times more likely to die compared to those who were euglycemic [(OR= 3.10, 95% CI (1.19 – 8.09) p=0.021]. Overall mortality was 22.4% within 48 hours of admission and risk of dying increase as the presence of poor clinical status add on.

Conclusion: Neonatal transportation in Dar es Salaam, Tanzania was observed to be challenging. Pre transfer care and monitoring during transportation was inadequate and this contributed to poor clinical status on admission. Hypothermia, hypoglycemia, hyperglycemia, hypoxia and poor perfusion on admission were associated with increased mortality.

Effective referral network is needed for improved neonatal health outcomes. Pre referral supportive care, transportation with improved monitoring and clear communication protocol should be invested and effectively utilized. Provision of warmth is critical during neonatal transfer using cost effective measures such as Kangaroo Mother Care.

Author Summary

Effective referral network is required between facilities to maintain continuum quality care for improved newborn health outcomes in particular the transportation gaps. This study has investigated pre referral care and transportation gaps in relation to admission clinical status and outcome of the neonates at the receiving facility. We found that pre referral documentation is poor, reflecting quality of care performed. Hypothermia, hypoglycemia, hyperglycemia, hypoxemia and poor perfusion at admission were associated with poor outcomes. All these are preventable conditions which if detected timely, managed and monitored effectively while neonates are being transported would have lesser impact on newborn health outcomes. There is a need to invest on referral network system at all levels and provide timely quality care for the newborn to arrive, survive, thrive and transform with no disability.

Introduction

Neonatal age is the most vulnerable period in human life with neonatal mortality contributing up to 47% of overall under-five deaths. About half of neonates die within the first 24 hours after birth and 75% in the first seven days of life (1). Most of these conditions, up to 75% are preventable through effective equitable measures such as early detection and timely management. Tanzania Demographic Health Survey (TDHS) of 2015-16 documented the current neonatal mortality of 25 deaths per 1000 live births, and it is challenging to reach the targeted Sustainable Development Goals (SDG 3.2) without investing in the continuum of care.

Transfer of the newborn whilst in a mother's womb is the safest method, but unforeseen complications during pregnancy and delivery cannot always be anticipated resulting in continuous need for transfer of these neonates from one facility to another. A well-established referral system is key to transfer neonates to a tertiary care facility (2–4).

Transportation of a neonate from one facility to another under ideal conditions is still a challenge in many developing countries, Tanzania included. Most of the neonates arrive in poor clinical conditions, which are mostly preventable. Previous studies have shown that hypothermia, hypoglycemia, poor perfusion and hypoxia are associated with increased mortality among transported neonates (5–9).

There is enough evidence to support that the transport by a skilled organized team reduces neonatal morbidity and mortality (6,10,11). The goal of all neonatal transport teams should be transporting a well-stabilized neonate. Pre transport stabilization is crucial; this entails securing the patency of the airway, breathing and circulation. Pre-transport procedures such as establishing an intravenous access should be carried out before arrival of the transport team (12)(13). Continuous monitoring need to be maintained on the way to a higher facility.

This study aimed at describing the transport characteristics (i.e. mode of transport, equipment, communication, accompanying personnel and monitoring enroute) used for the referred neonates. Additionally, it sought to assess the clinical status at admission in terms of the presence of hypothermia, hypoxia, prolonged capillary refill time and hypoglycemia for the transported neonates to Muhimbili National Hospital (MNH). Furthermore, it determined 48 hours' outcome and its associated factors in relation to the admission clinical status of referred neonates.

Ethics statement

Permission to carry out the study was sought from the Muhimbili University of Health and Allied Sciences (MUHAS) Institutional Review Board (IRB) with an approval number MUHAS-REC-07-2020-306. Written informed consent to participate in the study was sought from both the parent/guardian and health care personnel who escorted the neonate prior to any study procedure. Illiterate parents/ guardians were asked for a witness who participated within the discussion prior to obtaining their thumbprints and witness signature. All neonates received appropriate treatment according to the national treatment guidelines of Tanzania regardless of participation.

Materials and Methods

Study Area

Muhimbili National Hospital (MNH) is a tertiary referral centre and teaching hospital receiving patients from the five municipalities in Dar es Salaam (Ilala, Kinondoni, Kigamboni, Ubungo and Temeke) and other upcountry regions. It has a neonatal unit with a functioning Intensive Care Unit level 2 plus for caring critically ill neonates. The referral annual admissions range between 1440 and 3360 neonates.

Study Design

A descriptive cross sectional study with a longitudinal follow up was carried out at Muhimbili National Hospital (MNH), in Dar-es-Salaam region, Tanzania

Participant recruitment and data collection

All neonates referred to MNH from September 2020 to February 2021 were eligible for enrollment except for neonates with obvious congenital anomalies. A consecutive sampling was used to recruit referred neonates until the required sample size of 349 neonates was met. Sample size was calculated using the expected proportion of referred neonates transported to the neonatal unit of MNH who died in the first week post admission. This was established using a pilot study conducted at MNH prior to this, and was found to be 35%. A sample size of 349 was achieved using the Kish Leslie formula with 95% confidence level and 5% margin of error.

Data was collected day and night using a pretested structured questionnaire designed for the study (S1). Data on demographic characteristics of the study participants were obtained from the referral documents and by interviewing the caregiver or escorting personnel/nurse. The accompanying personnel was enquired about the transport process including pre transport care and monitoring during transport. All escorts were asked on whether they received a training on essential newborn care. Data on ambulance characteristics was obtained through direct observation of the transport by looking inside the transport for the presence of necessary equipment using WHO neonatal ambulance checklist (14). Clinical status on admission was measured using **TOPS** Model (15), whereby;

Temperature was recorded in degrees centigrade and Hypothermia - defined as an axillary temperature on admission < 36.5℃ measured with a digital thermometer (KONIG HC-DT10 Digital Thermometer, United Kingdom). Fever as axillary temperature on admission > 37.5℃.

Oxygen saturation (SPO2) was measured by using the pulse oximeter (ChoiceMMEd fingertip pulse oximeter, manufacturer: Beijing choice electronic technology Co., Ltd. Fuxing road A36 Beijing, 100039 China). Hypoxia was defined as oxygen saturation less than 90% in room air (16).

Perfusion, Capillary Refill Time was recorded by applying gentle pressure on the sternum for 3-5 seconds to cause blanching then the time taken for color to return was measured.

Sugar, Random Blood Glucose was measured by using a haemoglucometer machine (STANDARD™ GlucoNavii® GDH. Manufacturer: SD Biosensor, Inc. Gyeonggi-do, 16690, Republic of Korea). Hypoglycemia was defined as the blood glucose measurement of less than 2.5 mmol/L and Hyperglycemia as blood glucose measurement greater than 8.3 mmol/L.

All enrolled neonates were assessed at 48 hours post admission to capture the early outcome that was defined as survived or died.

Data Management and Statistical Analysis

Data analysis was done using SPSS version 23.0. Data for social-demographic and clinical characteristics of study participants were described using mean and standard deviations or medians and interquartile range for continuous variables. Proportions were presented in bar graphs. Multiple logistic regressions (odds ratio) was used to determine association with clinical outcome. A p-value ≤ 0.05 was considered statistically significant at 95% confidence interval.

Results

Baseline characteristics of the study participants

A total of 349 referred neonates were screened during the study period, one was excluded from the study due to a congenital anomaly which was incompatible with life (anencephaly). Figure 1 shows the proportion of participants received at Muhimbili National Hospital (MNH) stratified by

referring districts of Dar es Salaam, Tanzania. Out of the 348 neonates, 207 (59.5%) were term babies while 141 (40.5%) were preterm. Majority were male babies 197 (56.6%). 258 (74.1%) were delivered by Spontaneous Vertex Delivery (SVD) while 85 (24.4%) were born via caesarean section. The median gestation age was 38 weeks (IQR; 32 -39) with the mean birth weight of 2461 ± 936 g. Table 1 describes the socio- demographic and clinical characteristics of the study participants.

Pre Transport Care and documentation At Referring Facility

Two hundred and forty-six (79.9%) neonates had an intravenous line inserted prior to transfer. Temperature was measured in 176 (57.1%) neonates before transfer. Oxygen saturation and random blood glucose were measured pre transfer in only 143 (46.6%) and 116 (36.2%) neonates respectively.

Transport Process

Communication: Out of 348 neonates who were transferred to the neonatal unit of MNH, in only 26.7% the notification was given prior to transfer.

Escorting personnel: Out of the 340 neonates who were escorted, two hundred and twenty-four (65.9%) were escorted by the registered nurse. Seventy-three (21.5%) were escorted by the nurse attendant while eight (2.4%) were escorted by a doctor and thirty-two (9.4%) were escorted by the family member. Of the health care professional escorting the neonate, one hundred and sixty-nine (54.9%) had no training on essential newborn care.

Access to ambulance service: Majority of the neonates were transported by ambulance 308 (88.5%), followed by private car/tax 30 (8.6%), public service vehicle 9 (2.6%) and tricyclic motor vehicle 1 (0.3%).

Monitoring during transport: Monitoring was done to only 94 (27%) of the transferred neonates. Of those 69 (73.4%) had their temperature measured. Blood circulation was monitored in 37 (39.4%) of the transferred neonates while Random blood glucose was checked in 27 (28.7%). Just 57 (60.6%) had their oxygen monitored during transport.

Warmth during transport: Most of the referred neonates were kept warm using local clothes (Khanga/Kitenge), 233 (67.0%). Only 7 (2.0%) were kept on a Kangaroo Mother Care position, while no neonate was kept in an incubator during transfer as all ambulance were lacking incubators.

Reasons for referral: The main reason for transfer was to seek specialized care 164 (47.1%) followed by a lack of newborn unit 65 (18.7%), referred for further investigation 65 (18.7%), lack of equipment 23 (6.6%) and lack of personnel 7 (2.0%).

Ambulance Characteristics

For those who came with an ambulance 308, 294 (95.5%) had an oxygen supply, but in 42.5% of those the oxygen delivery system was not functioning. Only 201 (65.3%) had resuscitation equipment such as Ambu bag packed in the ambulances. Resuscitation drugs such as adrenaline were included in 200 (64.9%). Monitoring equipment were not available in 104 (33.8%) ambulances. IV fluids were present in 237 (77.0) while only 184 (59.7%) had suction apparatus. No ambulance had an incubator.

Figure 2 summarizes coverage gap during the referral journey of a newborn in Dar es Salaam, Tanzania.

Admission clinical status and outcome at the receiving facility, MNH

Seventy-eight neonates (22.4%) died within 48 hours of admission. On arrival, the clinical status of the neonates was as follows; 115 (33%) hypothermic, 74 (21.3%) hypoxic, 30 (8.6%) with poor perfusion and 49 (14.1%) hypoglycemic. Those with hypothermia (< 36.5^0C), were two times more likely to die compared to those who were normothermic (OR=2.09, 95% CI (1.05-4.20), p=0.037). Thirty-eight (50.7%) of those who had hypoxia (SPO2 < 90%) at arrival died compared to 37 (49.3%) of those who were not hypoxic (OR=2.88, 95%CI (1.44-5.74), p=0.003). Twenty-one (67.7%) neonates with prolonged capillary refill time (CRT) died compared to ten (32.3%) who survived (OR=4.76, 95%CI (1.80-12.58), p=0.002). Out of forty-nine neonates who arrived with hypoglycemia (RBG < 2.5mmol/l) twenty-one (42.9%) died in the first 48 hours (OR=2.13, 95%CI (0.96-4.74), p=0.064). Additionally, neonates who had hyperglycemia (RBG > 8.3mmol/l) on arrival were three times more likely to die compared to those who were euglycemic [(OR= 3.10, 95% CI (1.19 – 8.09) p=0.021]. Table 2 summarizes the results of univariate and multivariate analysis of factors associated with mortality.

The combined effect of poor clinical status on admission in terms of hypoglycemia, hyperglycemia, hypoxia, prolonged CRT and hypothermia were associated with increased mortality as shown in Figure 3.

Discussion

Despite its importance, neonatal transportation has often been overlooked in resource constrained and poor countries like Tanzania (13)(17,18). Due to lack of organized neonatal transportation system, most of these neonates arrive in poor clinical condition which influence their outcome negatively (2)(5). This study assessed the gaps on neonatal transportation, admission clinical status and outcome of the referred neonates in Dar es Salaam, Tanzania.

In our study, eighteen percent of the neonates were referred due to lack of a newborn unit in their facilities while almost half of others were referred for specialized medical or surgical care. Improving level of care in these referring institutions including establishment of well-equipped neonatal units will reduce a need for transfer. Having a dedicated neonatal transport team is also of paramount importance to ensure smooth transfer for those in need.

Pre transport stabilization and care is pivotal before transfer to a higher facility. It is generally agreeable that for a quality transport and desirable outcome, stabilization prior to transfer is mandatory (19). A study by Narang et al demonstrated an overall decrease in mortality among neonates in whom lifesaving interventions were done pre referral (20). In our study temperature was measured in more than half of the neonates prior to transfer but in the majority there was no documentation of what was done to those who were found to be hypothermic. In more than half of the referred neonates neither oxygen saturation nor Random blood glucose was measured or documented. Twenty percent of the neonates were transferred without a secured intravenous line which is vital for institution of drugs during resuscitation. Poor pre transport care contributed to poor conditions at arrival with subsequent poor outcome.

Three quarters of the referred neonates were brought in without any notification given to the neonatal unit that could give room for preparation and advice. This finding is similar to the study done by Abdulraheem et al whereby there was no communication or documented information

regarding the clinical stability of any of the neonates prior to transfer (13). In Tanzania referral system, the referring institution is tasked with providing the escorting personnel and most of the times they send staff who will render minimal interruption to the hospital running. Quite a number of neonates were escorted by nurse attendants 73 (21.5%) who were not trained to provide that level of care. Furthermore, this study found more than half of the health care personnel lacking training on essential newborn care. A study done in Jamaica reported one in three of the personnel accompanying the neonate were not skilled in neonatal resuscitation (12). Transfer of neonates by trained personnel has shown to reduce morbidity and mortality (21).

The preponderance of neonates who were referred to our facility came with hospital organized ambulances at 308 (88.5%) compared to other modes of transport. This is different from studies done in Ghana and Nigeria whereby majority of neonates were brought in by taxis and private vehicles at 36% and 43.9% respectively (18)(13). The findings are consistent with the study done in the EThekwini health district of KwaZulu-Natal South Africa whereby all the referrals were transported by ambulance (22). In this study despite a large number of neonates being brought in by ambulance, these ambulances were inadequate in equipment. Lack of equipment such as patient monitors impedes actions that could be taken to reverse deterioration during transport. It is not surprising that many neonates arrived at the tertiary hospital in unsteady clinical conditions (cold, hypoxic, hypoglycemic and hypothermic).

Most ambulances had oxygen supply similar to a study done in Bangladesh but in only 57.5% was the oxygen delivery system functioning (23). This fact plus lack of monitoring during transport clearly contributed to a number of neonates arriving hypoxic at the facility. A study by Pathak et al done in India using the TOPS score model established that neonates who had hypoglycemia and prolonged capillary refill time on admission had an overall poor outcome compared to their

counterparts (15). This is similar to our study which attested to the same. Additionally, we found a number of neonates who were hyperglycemic, with significant higher odds of mortality. These were the neonates whose blood sugar levels were not monitored and some were given continuous infusion of glucose without any blood glucose check.

Dar es Salaam has a tropical climate located 16m above sea level with an average annual temperature of 26.1^{0}C. Despite this fact, one third of the neonates were hypothermic on admission which was associated with increased mortality. This finding is similar to studies done in low resource settings in Ghana and India where almost half of the neonates were found to be hypothermic (18)(20). A study which was done on the same setting in 2003 by Manji et al found a threefold increase in mortality and morbidity among hypothermic neonates (8). Hypothermia in our study was contributed by lack of monitoring during transport and inferior means of maintaining warmth as more than two third of the referred neonates were covered by only light local clothes such as Khanga and Kitenge. Most facilities could not afford incubators in their ambulance, nevertheless the practice of Kangaroo Mother Care (KMC) which has been shown to be cheap and effective in preventing hypothermia was observed in only 2% of the transferred neonates. Rathod et al, in their study conducted in southern India had similar findings whereby KMC practice was not observed at all (17).

The overall mortality of neonates 48 hours post admission was 22%. This study demonstrated a correlation between poor clinical status on admission in terms of hypoxia, hypothermia, hyperglycemia and poor perfusion with increased risk of mortality 48 hours post admission. Overall, this study demonstrates inadequate pre transport care, documentation and stabilization, plus lack of monitoring enroute contributing to poor conditions of neonates on admission. Emphasis has to be placed on having well equipped ambulances dedicated to neonatal

transportation. Additionally, ensuring continuous training on health care workers on neonatal resuscitation and essential newborn care is key. Strengthening the referral system network remains a challenge to be tackled.

The study may have been exposed to recall bias as some of the information relied on the escorting health personnel. Hawthorne effect can't be ignored as the behaviors might have changed on the course of the study including improvement in the areas of referral and transportation of neonates as they become aware they are being observed. Despite the limitations, the findings reflect the gaps of referral system which need to be improved for quality of care of newborns at the primary and secondary levels. Further studies are required to explore and learn on barriers and facilitators of effective referral at all levels of care. All efforts should be done to ensure every small and sick newborn with a condition(s) that cannot be managed effectively with available resources should receive appropriate, timely referral through integrated newborn service pathways with continuity of quality care, including during transportation.

Acknowledgments

The authors acknowledge all staff at Muhimbili National Hospital, all caregivers/guardians of neonates, escorting persons and nurses who made this study possible to accomplish what we have. We are particularly obliged to Dr. Robert Moshiro and all members of Peadiatrics and Child Health Department at MNH and MUHAS. We would like to dedicate this work to our mentor, neonatologist, a father and a neonatal champion in Tanzania, the Late Dr. Augustine Massawe. May his soul rest in peace, Amin.

References

1. World health organization. Newborns: improving survival and well-being. Geneva; 2020.
2. Mir NA, Javied S. Transport of sick neonates: practical considerations. Indian Pediatr. 1989;26:755–64.

3. Kumar PP, Kumar CD, Venkatlakshmi A. Long distance neonatal transport- the need of the hour. Indian Pediatr. 2008;45(11):920–2.

4. Britto J, Nadel S, Maconochie I, Levin M, Habibi P. Morbidity and severity of illness during interhospital transfer: Impact of a specialised paediatric retrieval team. BMJ. 1995;

5. Segal S. Transfer of a premature or other high-risk newborn infant to a referral hospital. Pediatr Clin North Am. 1966;13(4):1195–205.

6. Chance GW, Matthews JD, Gash J, Williams G, Cunningham K. Neonatal transport: A controlled study of skilledassistance. Mortality and morbidity of neonates<1.5 kg birth weight. J Pediatr. 1978;93(4):662–6.

7. Gunn T, Outerbridge EW. Effectiveness of neonatal transport. Can Med Assoc J. 1978;118(6):646–9.

8. Manji KP, Kisenge R. Neonatal hypothermia on admission to a special care unit in Dar-es-Salaam, Tanzania: a cause for concern. Cent Afr J Med. 2003;49(3–4):23–7.

9. Dedeke I, Okeniyi J, Owa J, Oyedeji G. Point-of-admission neonatal hypoglycaemia in a Nigerian tertiary hospital: incidence, risk factors and outcome. Niger J Paediatr. 2011;38(2):90–4.

10. Agostino R, Fenton AC, Kollée LAA, Chabernaud JL, Carrapato MRG, Peitersen B, et al. Organization of neonatal transport in Europe. Prenat Neonatal Med. 1999;4(SUPPL. 1):20–34.

11. Sampathkumar PS, Gobinathan S. A study on status of neonatal transport to a level III neonatal intensive care unit. Int J Contemp Pediatr. 2018;5(3):1040.

12. Henry S, Trotman H. Challenges in neonatal transport in Jamaica: A resource-limited setting. J Trop Pediatr. 2017;63(4):307–13.

13. Abdulraheem MA, Tongo OO, Orimadegun AE, Akinbami OF. Neonatal transport practices in ibadan, Nigeria. Pan Afr Med J. 2016;24:1–10.

14. WHO. World health organization; Managing newborn problems, a guide for doctors, nurses and midwives. Geneva, Switzerland; 2003.

15. Mehta N, Sharma MK. Correlation of Acute Physiological Parameters With Immediate Outcome Among Neonates Transported To Special Care Newborn Unit: a Prospective Study. Indian J Child Health. 2018;05(04):280–3.

16. Morgan MC, Maina B, Waiyego M, Mutinda C, Aluvaala J, Maina M, et al. Oxygen saturation ranges for healthy newborns within 24 hours at 1800 m. Arch Dis Child Fetal Neonatal Ed. 2017;102(3):F266–8.

17. Rathod D, Adhisivam B, Bhat BV. Transport of sick neonates to a tertiary care hospital, south India: Condition at arrival and outcome. Trop Doct. 2015;45(2):96–9.

18. Tette EMA, Nuertey BD, Akaateba D, Gandau NB. The Transport and Outcome of Sick Outborn Neonates Admitted to a Regional and District Hospital in the Upper West Region of Ghana: A Cross-Sectional Study. Children. 2020;7(3):22.

19. Pan P. Inter Hospital Transfer of Critically Ill Neonates-Challenges Faced. J Pediatr Neonatal Care. 2017;6(2).

20. Narang M, Kaushik JS, Sharma AK, Faridi MM. Predictors of mortality among the neonates transported to referral centre in Delhi, India. Indian J Public Health. 2013;57(2):100–4.

21. Martínez Verónica R, López Gallo L, Rodríguez Medina D, Torre Gutiérrez M de la, Soto Mancilla JL, Márquez Amezcua M, et al. Transporte neonatal seguro en la población abierta del estado de Jalisco: impacto del programa S.T.A.B.L.E. en la morbilidad y mortalidad. Bol méd Hosp Infant Méx. 2011;68(1):34–9.

22. Ashokcoomar P, Naidoo R. An analysis of inter-healthcare facility transfer of neonates within the eThekwini Health District of Kwazulu-Natal, South Africa. South African Med J. 2016;106(5):514–8.

23. Baidya M, Shirin M, Saha LC. Transport Factors Affecting the Outcome of Referred Neonates Admitted in A Tertiary Care Hospital. Bangladesh J Child Heal. 2018;41(3):159–64.

Supporting Information Legend

Supportive Information 1(S1): Structured questionnaire English version

Figure Legends

Figure 1: Flow of the study participants referred from various districts of Dar es Salaam, Tanzania

Figure 2: Coverage gap during the referral journey of a newborn in Dar es Salaam, Tanzania

Figure 3: Combined effect of exposures on newborns mortality outcome at 48 hours post admission at Muhimbili National Hospital.

Tables

Table 1: Socio-demographic and clinical characteristics of study participants, N=348

Characteristic	Category	Frequency (n)	Percent (%)
**Sex**	Male	197	56.6
	Female	151	43.4
**Gestational age (weeks)**	<28	25	7.3
	28-32	68	19.7
	33-36	45	13.0
	>37	207	60.0
Median gestational age (week) (IQR)		38 (32-39)	
**Birth weight (g)**	Less than 1000	21	6.0
	1000-1499	37	10.6
	1500-2499	96	27.6
	2500+	194	55.8
Mean birth weight (SD) (g)		2461 (936)	
**Mode of delivery**	SVD	258	74.1
	Breech delivery	4	1.2
	Vacuum delivery	1	0.3
	Caesarean section	85	24.4
**Apgar score 1st and 5 minutes**	0-3 (low)	4	1.1
	4-6 (moderately abnormal)	65	18.8
	7-10 (reassuring)	279	80.1

Table 2: Univariate and Multivariate analysis of factors associated with mortality

Variable	Univariate analysis			Multivariate analysis		
	**cOR**	**95% CI**	**P -value**	**aOR**	**95% CI**	**P -value**
**Gestational age (weeks)**						
< 28	7.95	3.19 – 19.83	**< 0.001**	3.16	0.77 – 12.99	0.111
28 - 33	1.10	0.57 – 2.15	0.769	0.61	0.22 – 1.74	0.357
34 - 36	0.49	0.18 – 1.30	0.151	0.35	0.11 – 1.17	0.089
≥ 37	Ref					
**Weight (Kg)**						
< 1.0	4.65	1.86 – 11.59	**< 0.001**	1.99	0.45 – 8.74	0.362
1.0 – 1.4	3.03	1.43 – 6.41	**0.004**	2.54	0.70 – 9.24	0.158
1.5 – 2.4	1.07	0.57 – 2.02	0.829	1.56	0.68 – 3.60	0.293
≥ 2.5	Ref					
**Temperature (^{0}C)**						
Hypothermia (< 36.5)	3.65	2.04 – 6.53	**< 0.001**	2.09	1.05 – 4.20	**0.037**
Fever (> 37.5)	1.62	0.76 – 3.43	0.209	1.25	0.53 – 2.96	0.612
Normal (36.5 – 37.5)	Ref					
**Capillary Refill Time**						
Prolonged (> 3 seconds)	9.58	4.28 – 21.44	**< 0.001**	4.76	1.80 – 12.58	**0.002**
Normal (< 3 second)	Ref					
**Oxygen saturation**						
Hypoxia (< 90%)	5.98	3.41 – 10.51	**< 0.001**	2.88	1.44 – 5.74	**0.003**
Normal (≥ 90%)	Ref					
**Random blood glucose (mmol/L)**						
Hypoglycemia (< 2.5)	4.19	2.17 – 8.07	**< 0.001**	2.13	0.96 – 4.74	0.064
Hyperglycemia (> 8.3)	6.87	3.08 – 15.36	**< 0.001**	3.10	1.19 – 8.09	**0.021**
Normal (2.5 – 8.3)	Ref					

Key: cOR: crude odds ratio, aOR: adjusted odds ratio, Ref: Reference category

Figure
Click here to access/download;Figure;List of figures_Mpokigwa.docx

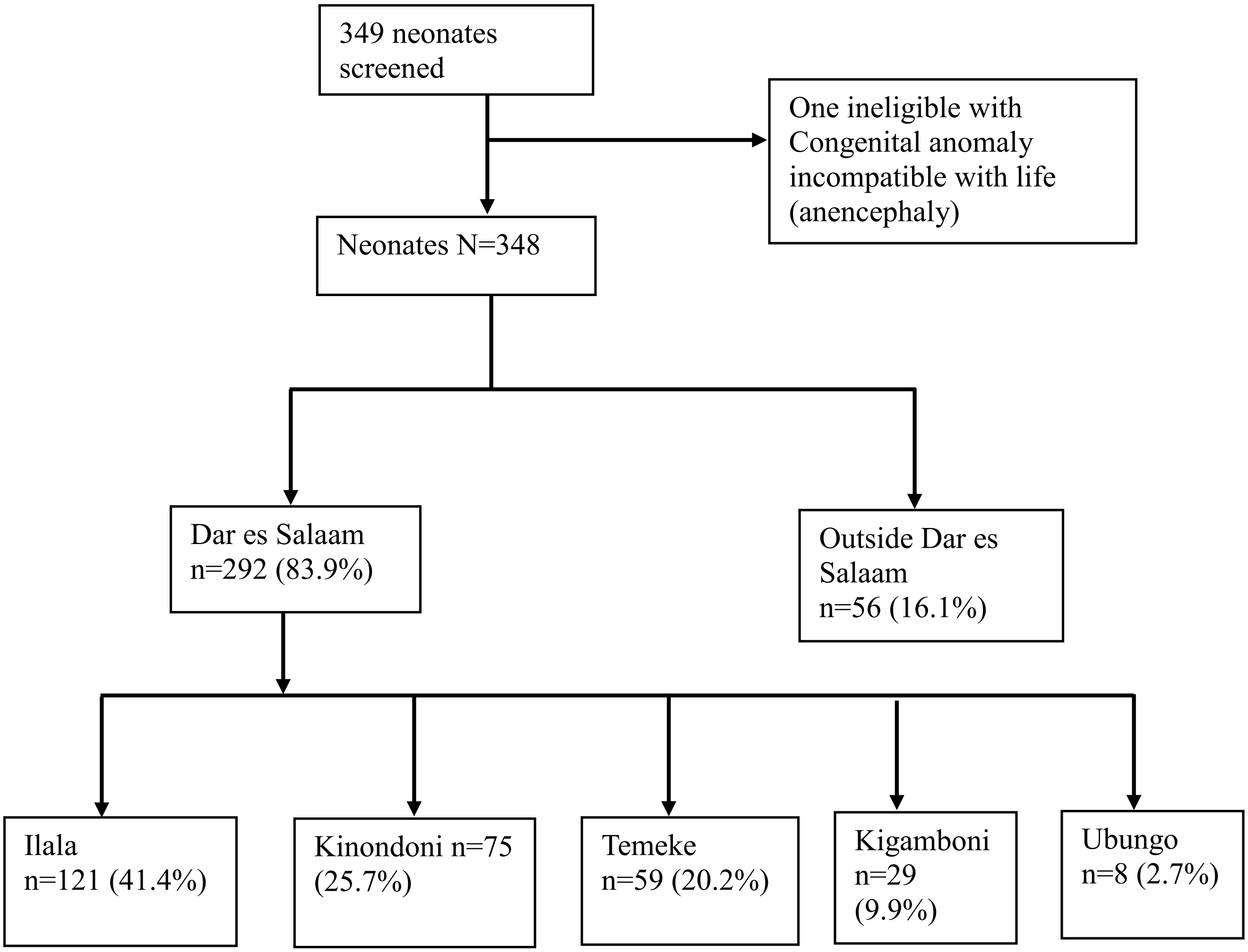

The % contribution were as follows:
Public hospital 89.4%
Private hospital 9.2%
Home 1.4%

Figure 1: Flow of the study participants referred from various districts of Dar es salaam,

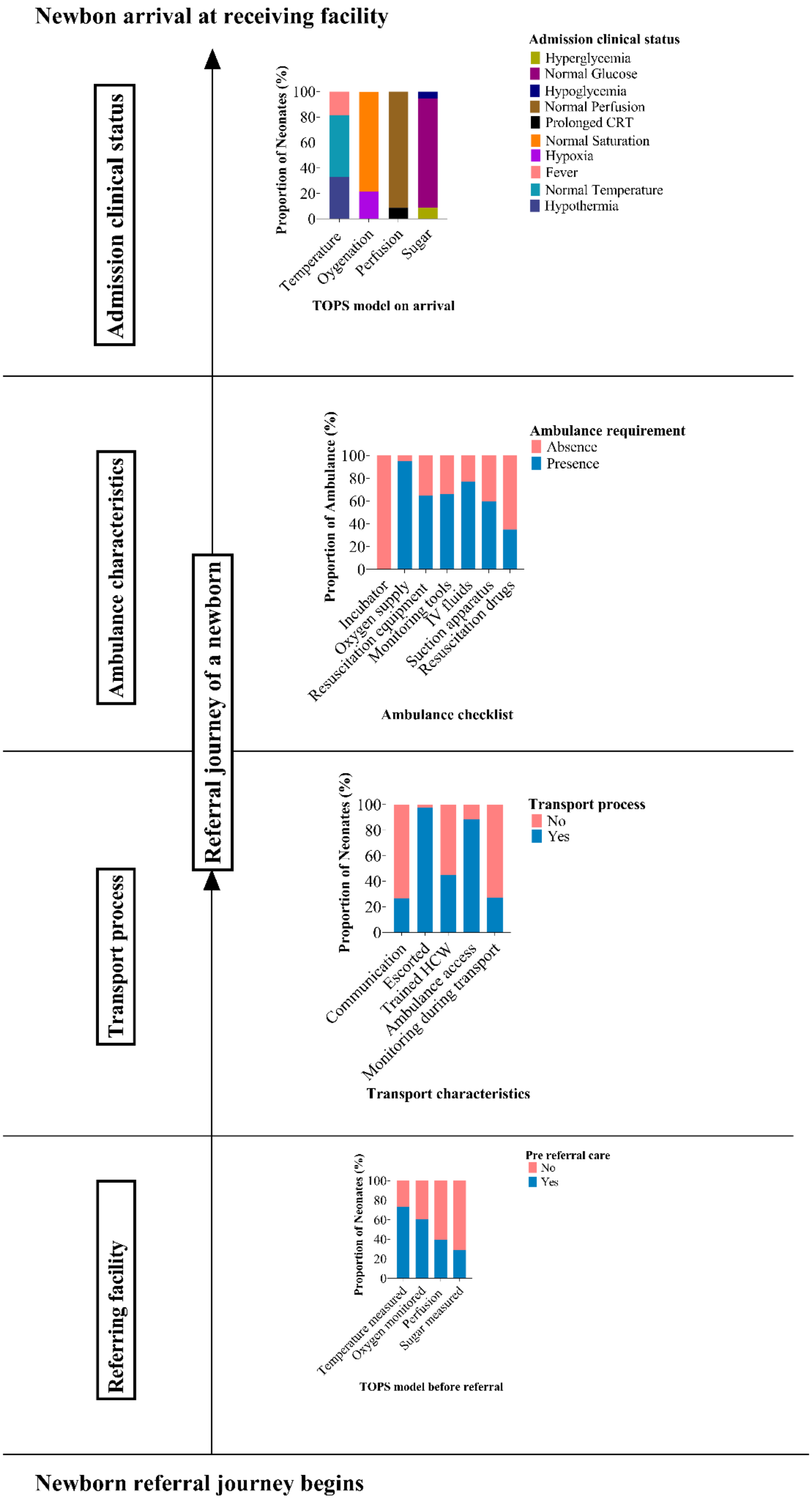

Figure 2.Coverage gap during the referral journey of a newborn in Dar es Salaam, Tanzania

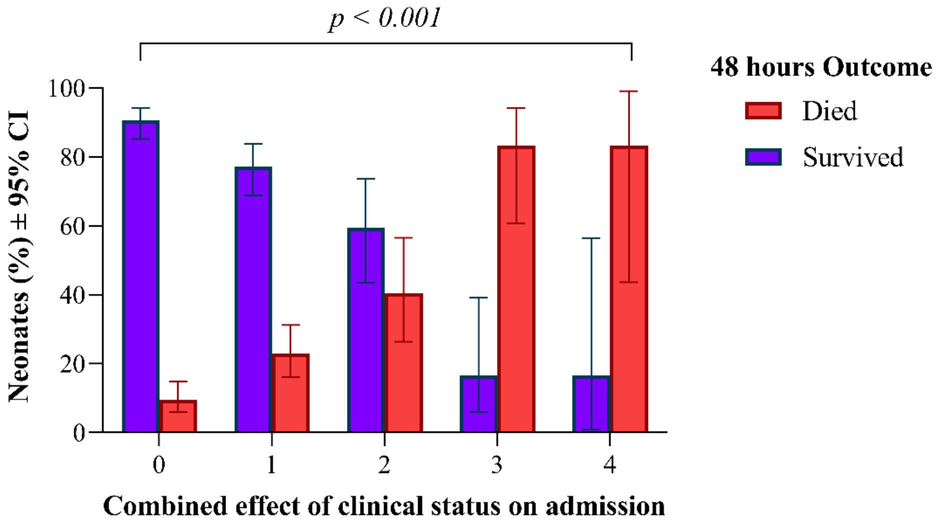

0 – Neonates without any poor clinical status on admission
1 – Neonates with one of the poor clinical status on admission
2 – Neonates with two of the poor clinical status on admission combined
3 – Neonates with three of the poor clinical status on admission combined
4 – Neonates with all four poor clinical status on admission combined

NOTE: Clinical status defined as presence or absence of Hypothermia, Hypoxia, Poor Perfusion, Hypoglycemia

Figure 3. Combined effect on newborns mortality outcome at 48 hours post admission at Muhimbili National Hospital

Click here to access/download
Supporting Information - Compressed/ZIP File Archive
Questionnaire English version.docx
