# Supplementary material for: Referral challenges and outcomes of neonates received at Muhimbili National Hospital, Dar es Salaam, Tanzania
Source: PLoS One. 2022 Jun 15;17(6):e0269479. doi: 10.1371/journal.pone.0269479 (PMC9200315; doi:10.1371/journal.pone.0269479)
Supplement: Attachment — Submitted filename: Response to reviewers.docx [file pone.0269479.s005.docx]

PONE-D-21-40535R1

Referral Challenges and Outcomes of Neonates Received at Muhimbili National Hospital, Dar es Salaam, Tanzania.

PLOS ONE

May 17, 2022

Dear editors,

We are grateful to have received your email dated 19^th^ April 2022, containing the reviewers’ comments pertaining our manuscript titled “Referral Challenges and Outcomes of Neonates Received at Muhimbili National Hospital, Dar es Salaam, Tanzania”

We are appreciative to the reviewers for taking their time to assess our manuscript a second time after our first review response. We have addressed all the concerns raised by the independent peer reviewers and improved the overall quality of our manuscript.

Please find below point-by-point response, clearly indicating how and where in the manuscript changes have been made (line numbers). To assist you in readily tracking our amendments, we included a marked up copy of the manuscript that highlights changes made to the original version. We also enclose a “clean” version of the revised manuscript. All comments from the reviewers (previous and current comments) have been integrated into the manuscript itself (not only in the response letter). Study limitations, conclusion and recommendations revised accordingly. Reviewers’ responses are highlighted in the manuscript.

Additional edits to reformat the manuscript have been done and can be observed with track changes and summarized in the response to comments letter specifying sections and line numbers as per no. 8 below. Kindly note that, these edits do not alter any information as per reviewers comments but rather improve the organization and grammar of the manuscript.

We very much hope that this revised version of the manuscript will now be suitable for publication in PLOS medicine. We look forward to hearing from you.

Yours Sincerely,

Dr Mpokigwa Kiputa

**Journal Requirements:**

Please review your reference list to ensure that it is complete and correct. If you have cited papers that have been retracted, please include the rationale for doing so in the manuscript text, or remove these references and replace them with relevant current references. Any changes to the reference list should be mentioned in the rebuttal letter that accompanies your revised manuscript. If you need to cite a retracted article, indicate the article’s retracted status in the References list and also include a citation and full reference for the retraction notice.

Thank you for the comment, reference list has not been updated and remains as per previous edits according to initial reviewer’s comments.

**Reviewer #1**: All my comments have been addressed. I would like to congratulate the authors for a good work done.

Thank you for the congratulatory note. We really do appreciate your time invested to improve the quality of the manuscript.

**Reviewer #4:** Dear authors, Thank you for addressing my queries in detail and submission of revisions. Authors have improved the manuscript very nicely, but I have some minor issue:

Thank you for the positive remarks

1. Although grammar part had been improved but please look on use of unnecessary capital words. Line 166: Model, line 185: Spontaneous Vaginal Delivery.

Thanks for noticing. The capitalization errors have been addressed accordingly in line 169 and line 188 in the manuscript. The whole manuscript was revised and other font edits have been made in line 146, 147 and 158.

2. Main issue is with the inadequacy of the data analysis for primary aim of the study.

   In abstract part (background section, line 24): Authors claim “This study assessed the pre-referral care……”

   However, as per the information provided in result section (line 189), no details of pre referral care have been given by the authors. The information just describes the numbers of neonates having IV-line insertion, temperature measurement, oxygen saturation and blood glucose monitoring.
   Same point had been highlighted in the response letter points number 3 and 6 regarding inadequacy of documentations in referral cards.

   I will suggest author to add this point (i.e. inadequate assessment of pre referral care) in limitations sections, please.

Pre referral care entails stabilization of the sick neonate prior to transfer according to the S.T.A.B.L.E (Sugar, Temperature, Airway, Blood pressure, Lab work and Emotional support) program. It was developed by Kristine Karlesen in 1980s as part of research project. Henceforth adopted worldwide as a standard practice.

Establishing an IV access, temperature measurement, oxygen saturation and blood glucose monitoring is part of the pre referral care as it was laid out in our study using the TOPS model. Although we agree that to a certain extent it was inadequate given the fact that not all parameters were assessed and no direct assessment was carried out at the referring centers. In this study, pre referral care assessment was based on documentation provided from the referral notes.

In limitations section line 347-349, this point has been added on and it now reads as follows;

Limitation in recording full data due to poor/incomplete documentation based on the referral forms, consequently contributed to the inadequate data of pre-referral care assessment, reasons for referral and monitoring enroute information

In addition, the methodology section in the abstract has been updated accordingly, please refer to line 29, 30 and 34 to match line 137 -139 in the main manuscript (highlighted yellow for easier tracing)

3. In abstract part, result section line 44-49 need to be rephrased while reporting odd’s ratios and p-value. Please avoid repeated use of odds of dying or likely to die repeatedly as this can be easily understandable from odd’s ratio against each variable

The statement in result section line 45-51 has been rephrased and it now reads as follows;

Hypothermic neonates had an increased chance of dying compared to those who were normothermic (OR=2.09, 95% CI (1.05-4.20), p=0.037). The chance of dying among those presenting with hypoxia was almost three times (OR=2.88, 95%CI (1.44-5.74), p=0.003) while those with poor perfusion was almost five times (OR=4.76, 95%CI (1.80-12.58), p=0.002). Additionally, neonates who had hyperglycemia (RBG > 8.3mmol/l) on arrival had a higher probability of dying compared to those who were euglycemic [(OR= 3.10, 95% CI (1.19 – 8.09) p=0.021].

4. In conclusion part, line 58-59; please shift this line from conclusion to discussion part. This is not a finding of this study but may serve as future recommendation.

We agree with this observation. The line aforementioned has been removed from the conclusion part and added to recommendation line 355-356 in the main manuscript

5. Line 213: I request to the authors to state indications of referral more explicitly rather than mentioning “seek specialized care, lack of newborn unit or lack of equipment”, which are very vague.

The indications for referral were extracted from the referral notes at the time of admission before any further history was taken. Unfortunately, the reasoning were clustered and no much information provided. This has been addressed as one of the limitations of our study. Documentation using standard harmonized forms is a critical and currently NEST 360 program is working on improving all forms including introducing inpatient neonatal register.

The study limitation has been updated to accommodate this information and it reads as

Limitation in recording full data due to poor/incomplete documentation based on the referral forms, consequently contributed to the inadequate data of pre-referral care assessment, reasons for referral and monitoring enroute information. Please refer to line 347 – 349.

6. As authors have acknowledged in response letter at points number 3, 6 and 9th, there are some limitations in recording full data. So, please add these statements in the limitations section at the end of manuscript.

The statement has been added accordingly in line 347-349 in the limitation section. It reads as follows;

Limitation in recording full data due to poor/incomplete documentation based on the referral forms, consequently contributed to the inadequate data of pre-referral care assessment, reasons for referral and monitoring enroute information. Please refer to line 344 – 346 in the manuscript.

7. Please avoid repetition of the facts “no ambulance had an incubator” at line 211-212, line 222.

The aforementioned line 211 which now stands as line 214 had been modified accordingly to avoid repetitions. The edits have been made in the manuscript.

8. Additional edits of grammar, font and some wordings have been made in the manuscript for the purpose of clarity. They are on track changes and summarized per section and line numbers below. All the edits are cosmetic and do not alter previously revised manuscript.

Abstract – line 26, 34, 41, and 60

Introduction – line 105

Materials and Methods - line 120, 123, 124, 146, 147, 158, 169, 175 and 177

Results – 185, 192, 193 and 228

Discussion – 251, 275, 285, 290, 302, 305, 325, 326, 339, 352 and 354
